# Utilization and Dose Optimization of Angiotensin-Converting Enzyme Inhibitors among Heart Failure Patients in Southwest Ethiopia

**DOI:** 10.1155/2019/9463872

**Published:** 2019-04-24

**Authors:** Yirga Legesse Niriayo, Kabaye Kumela, Kidu Gidey, Mulugeta Tarekegn Angamo

**Affiliations:** ^1^Department of Clinical Pharmacy, School of Pharmacy, College of Health Sciences, Mekelle University, Mekelle, Tigray, Ethiopia; ^2^Department of Clinical Pharmacy, School of Pharmacy, Faculty of Health Sciences, Jimma University, Jimma, Oromyia, Ethiopia; ^3^Division of Pharmacy, School of Medicine, University of Tasmania, Australia

## Abstract

**Background:**

Optimal use of angiotensin-converting enzyme inhibitors (ACEIs) is crucial to improve the treatment outcome in heart failure patients. However, little is known about the optimal use of ACEIs among heart failure patients in our setting. Therefore, our study aimed to investigate the utilization and optimal dosing of ACEIs and associated factors in heart failure patients.

**Method:**

A cross-sectional study was conducted on randomly selected patients with heart failure between February 2016 and June 2016 at ambulatory care clinic of Jimma University Medical Center, Ethiopia. Data were collected through patient interview and review of medical records. Binary logistic regression analysis was done to identify factors associated with utilization and optimal dosing of ACEIs.

**Results:**

A total of 308 patients were included in the final analysis of this study. The mean (±standard deviation) age of the patients was 52.3 ±15.5 years. Out of the total, 74.7% of the patients were receiving ACEIs. Among the patients who were receiving ACEIs, only 35.7% were taking optimal dose. New York Heart Association (NYHA) class III (Adjusted odds ratio (AOR):0.12, 95% confidence interval (CI):0.02–0.98), valvular heart disease (AOR: 0.27, 95% CI: 0.13-0.56), hypertension (AOR: 5.82, 95% CI: 2.16-15.71), and diabetes mellitus (AOR: 3.84, 95% CI: 1.07-13.86) were significantly associated with the use of ACEIs, whereas age ≥65 (AOR: 2.61, 95%CI: 1.20-5.64), previous hospitalization for heart failure (AOR: 2.08, 95%CI: 1.11-3.92), diuretic use (AOR: 5.60, 95%CI: 2.75-11.40), and dose of furosemide >40mg (AOR: 9.80, 95%CI: 3.00-31.98) were predictors of suboptimal dosing of ACEIs.

**Conclusion:**

Although majority of patients were receiving ACEIs, only about one-third were using optimal dosage. Valvular heart disease and NYHA class III were negatively associated with the use of ACEIs while previous hospitalization for heart failure, old age, diuretic use, and diuretic dose were predictors of suboptimal dosing of ACEIs. Therefore, more effort needs to be done to minimize the potentially modifiable risk factors of suboptimal use of ACEIs therapy in heart failure patients.

## 1. Introduction 

Heart failure (HF) is a global public health threat that affects about 26 million people worldwide [1]. Currently, HF becomes one of the most important public health concerns in developing countries including sub-Saharan Africa [2, 3]. HF is a debilitating illness that is associated with a high burden of morbidity and mortality, impaired quality of life, and increased health care expenditure [1–3]. Despite the major therapeutic advances that have occurred in the management of HF patients over the past decades, HF remains the leading cause of morbidity, mortality, and economic burden for health care budgets [4].

Studies have shown that the implementation of evidence-based guideline-recommended drug treatments for HF has resulted in the reduction of HF associated morbidity and mortality [4, 5]. However, HF remains a substantial contributor of morbidity and mortality due to the complexity of multiple comorbidities, polypharmacy, advanced age, and lack of implementation of recommended medications or proper titration of these drugs [4, 5].

Angiotensin-converting enzyme inhibitors (ACEIs) are the cornerstone of standard HF therapy. In absence of contraindication, ACEIs should be prescribed for all patients with systolic HF [6, 7]. However, they are often underutilized in a real clinical practice [8, 9]. ACEIs have been proved to have mortality and morbidity benefits in patients with systolic HF in several clinical trials [10–13]. In HF patients with reduced ejection fraction, ACEIs therapy leads to symptomatic improvement, reduced hospitalization, and enhanced survival [10, 14].

The clinical benefits of ACEIs in HF patients appear to be dose-dependent and a better benefit has occurred at higher target doses [15, 16]. ACEI uptitration to a maximum tolerable dose is important in chronic HF patients to reduce the incidence of hospitalization, morbidity, and mortality as well as improve the quality of life of the patients [17–19]. Several studies indicated that a target dose of ACEI is achievable in the majority of chronic HF patients and the achieved optimal dose was associated with better treatment outcomes [4, 14, 17, 18]. Therefore, every effort should be made to achieve the target dose or maximum tolerable dose to get the maximum clinical benefit [6, 7].

According to the evidence-based guidelines [6, 7], the recommended daily target doses of ACEIs are 20–40 mg enalapril, 10 mg Ramipril, 150 mg captopril, 20–40 mg Lisinopril, 40mg fosinopril, 4mg trandolapril, 40 mg quinapril, or 8-16 mg perindopril. Although evidence-based guidelines recommend the use of the target doses used in clinical trial for the treatment of HF patients [20, 21], attaining target doses is often challenging in daily practice owing to the multiple comorbidities, polypharmacy, and side effects particularly in older individuals [22]. Generally, ACEIs are prescribed in doses lower than the target doses that have been shown to reduce morbidity and mortality in patients with HF in clinical trials [9, 19].

The target doses of ACEIs are tolerable in the majority (>80%) of HF patients if titrated appropriately [17, 21, 23]. However, several studies reported that substantial number of patients were receiving below the target doses of ACEIs [15, 22, 24]. More importantly, underutilization and suboptimal dosing of ACE inhibitors limit the morbidity and mortality benefit for HF patients [18, 19]. To our knowledge, however, the optimal use of ACEIs has not been investigated yet in our healthcare settings. Hence, our study investigated the optimization of ACEIs therapy and associated factors in the management of ambulatory patients with HF.

## 2. Material and Methods 

### 2.1. Study Design and Study Setting

A hospital-based cross-sectional study was conducted from February 2016 to June 2016 at ambulatory care clinic of Jimma University Medical Center, which is the major public hospital in southwest Ethiopia with a catchment population of about 15 million people.

### 2.2. Study Population and Data Collection Procedure

Adult patients (age ≥18 years) with a diagnosis of HF and a baseline ejection fraction <40% confirmed by Echocardiogram and those who had been on regular follow-up for at least 6 months were included in the study. Patients with precautions and contraindications to the use of ACE inhibitors including pregnancy, history of angioedema, allergic reaction to the drug, dry cough, hyperkalemia (> 5.5 mEq/mL), hypotension (<90/60mmg), renal insufficiency (creatinine clearance <30 mL/ min), aortic or mitral stenosis, and bilateral renal artery stenosis and those patients with incomplete medical record were excluded from the study.

A sample of 340 was calculated using a single population proportion formula assuming 50% proportion of ACE inhibitor utilization and dose optimization among patients with HF, 95% confidence level, 5% margin of error, and 10% of contingency for nonresponse rate. From a total of 340 participants approached, 32 patients were excluded from the study due to contraindication to ACEIs [25] and incomplete medical record [7]. Patients were recruited randomly into the study during their appointment for medication refilling. Data concerning sociodemographic were retrieved by interviewing patients using the standardized questionnaire. The clinical and treatment related characteristics were retrieved from patients' medical record using data abstraction checklist.

We determined the utilization and dose of ACEIs. ACEIs were said to be underutilized if they were not used by the patients in absence of contraindications. The optimal dosing of ACEISs was determined according to the evidence-based guideline-recommended target doses [21]. Accordingly, the dose of ACEIs was said to be optimal if it was given at guideline-recommended target dose or a maximum tolerable dose is given for the patients. For enalapril, the optimal dose was considered if the dose was ≥20 mg daily or a maximum tolerable dose and for captopril, the dose was said to be optimal if it was ≥150 mg daily or a maximum tolerable dose, whereas the dose was said to be suboptimal if the patient was taking any dose of ACEIs below the target dose in absence of contraindications for uptitration.

### 2.3. Data Analysis

EPI data management (version 4.2.0) and the Statistical Package for the Social Science (SPSS version 21.0) were used to record and analyze the data, respectively. Descriptive analysis was computed using frequency for categorical variables. Moreover, the mean (standard deviation) and median (interquartile) were computed for continuous variables. Multicollinearity was checked to test correlation among independent variables using variance inflation factor and none was collinear. Univariable logistic regression analysis was performed to determine the association of each independent variable with utilization and dose optimization of ACEIs, and then independent variables with p value* <*0.2 in univariable analysis were included in the multivariable binary logistic regression model to identify predictors of treatment optimization of ACEIs. A p value of <0.05 was considered statistically significant in all analyses.

### 2.4. Ethics Approval and Consent to Participate

Approval for this study was obtained from institutional review board of Jimma University, College of Health and Medical Sciences. The aim and protocol of the study were fully explained to all study participants included in the study and written informed consent was obtained from each participant. The personal information was entirely confidential and protected. All methods were performed in accordance with the approved institutional guidelines.

## 3. Results

### 3.1. Sociodemographic and Clinical Characteristics

A total of 308 patients were included in this study. The mean [± standard deviation (SD)] age of the patients was 52.3±15.5 years and 51.5% were males. The majority (63.3%) of the patients were rural dwellers and more than half (56.5%) were unable to write and read (Table 1).

### 3.2. Clinical and Medication-Related Characteristics

More than half (57.1%) of the patients had been hospitalized one or more times in the preceding one year. The majority of patients were in NYHA class III (48.4%) and II (44.5%). Almost two-thirds (62.7%) of the patients had two or more comorbidities and the most commonly identified comorbidity was ischemic heart disease (45.1%) followed by hypertension (26.9%) and valvular heart disease (21.4%). The mean (±SD) number of medications per patient was 4.2±1.2 and 43.5% of the patients were taking five more medications. Among the HF medications, the most frequently used medications were loop diuretics (78.9%) followed by ACEIs (74.7%) and beta-blockers (61 .4%) (Table 2).

### 3.3. Utilization and Dosing of ACEIs in HF Patients

Out of the total, 230 (74.7%) patients were receiving ACEIs. Enalapril (93.5%) and captopril (6.5%) were the only prescribed ACEIs. Among the patients who were receiving ACEIs, only 82 (35.7%) were taking optimal dose. Overall, almost three-fourths (73.4%) of the patients were either not receiving ACEIs or receiving a suboptimal dose. The mean daily doses of enalapril and captopril that were taken by HF patients were 12 mg and 55 mg, respectively (Table 3).

### 3.4. Factors Associated with the Use of ACEIs

Univariable logistic regression analysis was performed to compare HF patients who were taking ACEIs and not taking ACEIs using the sociodemographic, clinical, and treatment related characteristic. Accordingly, NYHA class III (P=0.047) and valvular heart disease (P=0.001) were negatively associated with the use of ACEIs while hypertension (P=0.001) and diabetes mellitus (P=0.040) were positively associated. Moreover, variables with P<0.2 in the univariable were reentered into the multivariable logistic regression model. The whole model containing all predictors was statistically significant (Chi-square = 105.057, df = 9, P<0.001). According to multivariable logistic regression analyses, NYHA class III (Adjusted odds ratio (AOR):0.12, 95% confidence interval (CI):0.02–0.98), valvular heart disease (AOR: 0.27, 95% CI: 0.13-0.56) hypertension (AOR: 5.82, 95% CI: 2.16-15.71), and diabetes mellitus (AOR: 3.84, 95% CI: 1.07-13.86) were significantly associated with the use of ACEIs (Table 4).

### 3.5. Factors Associated with Dose Optimization of ACEIs

Factors associated with optimal dosing of ACEIs were also identified using univariable and multivariable regression model. The whole model containing all predictors was statistically significant (Chi-square = 57.059, df = 7, P<0.001). Age ≥ (AOR: 2.61, 95%CI:1.20-5.64), previous hospitalization for HF (AOR: 2.08, 95%CI: 1.11-3.92), diuretic use (AOR: 5.60, 95%CI: 2.75-11.40), and dose of furosemide >40 mg (AOR:9.80, 95%CI:3.00-31.98) were significantly associated with suboptimal dose of ACEIs (Table 5).

## 4. Discussion 

Despite multiple drug therapies, HF remains the leading cause of morbidity and mortality [4]. Optimization of HF therapy results in a significant reduction of morbidity and mortality associated with HF [25, 26]. Assessment of treatment optimization of ACEIs is crucial to provide important information for clinicians working in the management of chronic HF. Therefore, our study determined the utilization and dose optimization of ACEIs and associated factors among patients with HF. Accordingly, ACEIs were either underutilized or under-dosed in the majority (73.4%) of the HF patients.

In agreement with Palestine study [9], the majority of the patients were either not receiving ACEIs or not receiving optimal dose of ACEIs in absence of contraindication to the use or to increase the dose to optimal dose. The use of ACEIs in systolic HF conferred a 16-20 % reduction in mortality [27]. Although evidence-based guidelines recommend the use of ACEIs in all patients with systolic HF [6, 7, 21], about one-fourth of the patients were not receiving ACEIs without known reason in our study. Consistent with our finding, ACEIs were often underutilized in other similar studies [5, 8, 9, 28].

The use of ACEIs was negatively associated with NYHA class III in our study which was also supported by other study [21]. The presence of valvular heart disease was also negatively associated with the use of ACEIs. This could be due to the controversial indication of ACEIs in valvular heart disease [29, 30]. Patients with hypertension were more likely to use ACEIs which was in line with Palestine study [9]. In addition, the presence of diabetes mellitus was positively associated with the use of ACEIs. The possible justification for the positive association of hypertension and diabetes mellitus with the use of ACEIs might be due to the additional indication and renoprotective effect of ACEIs in hypertension and diabetes mellitus [31].

Studies have demonstrated a dose-related clinical benefit of ACEI therapy in HF patients [32] and a higher dose was associated with a better treatment outcome [22]. Hence, evidence-based guidelines recommend uptitration of ACEIs to a target dose unless there is tolerability problem [6, 7, 21]. In contrast, almost two-thirds (64.7%) of patients were on suboptimal dose of ACEIs in this study. This could be attributed to the absence of heart HF guideline in our setup.

In agreement with our study, the majority of the patients were below the target dose in other similar studies [5, 15, 33, 34]. In contrast, our finding was quite different from a study in Germen [35] in which 62% of the ACEIs doses were at the guideline-recommended target dose. This might be due to the difference in medical practitioners' expertise and the poor awareness of dose titration practice of ACEIs as observed in this study.

In the present study, suboptimal dosing of ACEIs was significantly associated with previous hospitalization which is in line with other similar studies [18, 32]. Older patients (Age ≥65) were more likely to receive a suboptimal dose of ACEI compared to their counterparts (age<65). However, studies have revealed that optimal dose of ACEIs is associated with reduced all-cause five-year mortality in very old patients with systolic HF [13]. Despite optimal dose is achievable in the majority older patients with HF [13], 80% of the older patients were receiving a suboptimal dose of ACEIs in the present study. This could be explained that medical practitioners may fail to prescribe high dose of ACEIs for older patients due to fear of intolerance.

Appropriate use of diuretics affects the success of other medications given for the treatment of HF. Studies have shown that the use of diuretics increased with age and the increased use of diuretics was associated with a decrease in the use of the recommended HF drugs including ACEIs and beta-blockers [36]. More importantly, high dose of diuretics usually limits the uptitration of drugs that have survival benefit in HF patients including ACEI [37]. Consistent with this, the diuretic use and dose of furosemide were significantly associated with the suboptimal dose of ACEIs. This could be explained that excessive diuresis can increase the risk of hypotension and renal insufficiency with ACE inhibitors due to volume depletion [37, 38]. Therefore, clinicians should prescribe diuretics with careful consideration taking into account their negative effect on uptitration of other drugs. Particularly, the dose of diuretics needs to optimized to allow titration of ACEIs to target dose.

Our study has some limitations. The cross-sectional nature of our study may not provide adequate evidence of causality regarding the suboptimal use of ACEIs and its contributing factors. Our study should be extrapolated to other countries with caution as the finding of this study depends on the difference in population demographics, disease distribution, clinician's expertise, and the health care system.

## 5. Conclusion 

Although majority of patients were receiving ACEIs, only about one-third were using optimal dosage. While hypertension and diabetes mellitus were positively associated with the use of ACEI, the presence of valvular heart disease and NYHA class III were negatively associated. Moreover, previous hospitalization for HF, old age, diuretic use, and dose were significantly associated with suboptimal dosing of ACEIs. We suggest the implementation of multidisciplinary team approach including clinical pharmacists in the medication review and patient monitoring process at ambulatory care clinics for the optimization of ACEIs and achieving definite outcomes in patients with HF. In addition, more efforts need to be made to minimize potentially modifiable risk factors of suboptimal use of ACEIs in HF patients.

## Figures and Tables

**Table 1:** Sociodemographic related characteristics of HF patients (n=308).

Characteristics	n (%)
Sex, male	157 (51)

Age in years	

<65	227 (73.7)

≥65	81 (26.3)

Residence, rural	192 (63.3)

Educational level	

Unable to write and read	174 (56.5)

Primary education	52 (19.6)

Secondary education	26 (8.4)

College and above	56 (18.2)

Marital status	

Married	233 (75.6)

Single	3 (9.7)

Divorced	19 (6.2)

Widowed	26 (8.4)

**Table 2:** Clinical and medication related characteristics of HF patients (n=308).

Characteristics	n (%)
Previous hospitalization	

no	132 (42.9)

Yes	176 (57.1)

Duration	

<2	108 (35.1)

>=2	200 (64.9)

NYHA class	

I	22 (7.1)

II	137 (44.5)

III	149 (48.4)

Frequently identified comorbidities	

Ischemic heart disease	139 (45.1)

Hypertension	83 (26.9)

Valvular heart diseases	66 (21.4)

Atrial fibrillation	64 (20.8)

Diabetes mellitus	52 (16.9)

Hyperthyroidism.	22 (7.1)

Chronic kidney disease	17 (5.5)

Number of comorbidities	

<2	115 (37.3)

≥2	193 (62.7)

Number of medications	157 (51)

<5	174 (56.5)

≥5	144 (43.5)

Commonly used medications	

Loop diuretics	240 (78.9)

ACEIs	230 (74.7)

Beta-blockers	189 (61.4)

ACEIs + Beta-blockers	180 (58.4)

Antiplatelets	187 (60.7)

Statins	134 (43.5)

Potassium sparing diuretics	68 (22.1)

Cardiac glycosides	58 (18.8)

ACEIs, angiotensin converting enzyme inhibitors; NYHA, New York Heart Association.

**Table 3:** Type and dose of ACEIs used in HF patients (n=230).

Variables	Medications	
	Enalapril	Captopril
Number of patients on the medication (%)	215 (93.5)	15 (6.5)

Number of patients on the optimal dose (%)	81 (37.7)	1 (6.7)

Number of patients on 50 to <100% of the target dose (%)	25 (11.6)	5 (33.3)

Number of patients on <50% of the target dose (%)	109 (50.7)	9 (60)

Mean (SD) daily dose (mg/d)	12 (8.3)	55 (33.6)

Median (IQR) dose received (mg)	7.5 (5-20)	37.5 (37.5-75)

Minimum dose used (mg/d)	2.5	18.75

Maximum dose used (mg/d)	40	150

SD, standard deviation; IQR, interquartile.

**Table 4:** Factors associated with the utilization of ACEIs (n=308).

Variables	ACEIs use		COR (95% CI)	p-value	AOR (95%CI)	p-value
	No, n (%)	Yes, n (%)				
Age category						

<65	62 (79.5)	165 (71.7)	1	1	1	1

>=65	16 (20.5)	65 (28.3)	1.53 (0.82-2.84)	0.181	1.02 (0.47-2.19)	0.964

Hospitalization in the last one year						

No	39 (50.0)	93 (40.4)	1	1	1	1

Yes	39 (50.0)	137 (59.6)	1.47 (0.88-2.47)	0.141	1.33 (0.69-2.55)	0.394

Number of comorbidities						

<2	36 (46.2)	79 (34.3)	1	1	1	1

>=2	42 (53.8)	151 (65.7)	1.64 (0.97-2.76)	0.064	1.02 (0.51-2.01)	0.964

NYHA class					.	

I	3 (3.75)	19 (8.3)	1	1	1	1

II	12 (15)	125 (54.8)	0.47 (0.10-2.15)	0.331	1.11 (0.13-9.55)	0.925

III	65 (81.3)	84 (36.8)	0.19 (0.04-0.83)	0.027	0.12 (0.02-0.98)	0.047

Ischemic heart disease						

No	48 (61.5)	121 (52.6)	1	1	1	1

Yes	30 (38.5)	109 (47.4)	1.44 (0.85-2.44)	0.172	1.23 (0.60-2.51)	0.579

Hypertension						

No	72 (92.3)	153 (66.5)				

Yes	6 (7.7)	77 (33.5)	6.04 (2.51-14.51)	P<0.001	5.82 (2.16-15.71	0.001

Valvular heart disease						

No	42 (53.8)	199 (86.5)	1	1	1	1

Yes	36 (46.2)	31 (13.5)	0.18 (.10-0.32)	P<0.001	0.27 (0.13-0.56)	0.001

Diabetes mellitus						

No	75 (96.2)	181 (78.7)	1	1	1	1

Yes	3 (3.8)	49 (21.3)	5.91 (1.78-19.63)	0.004	3.84 (1.07-13.86)	0.040

NYHA, New York Heart Association, ACEIs, angiotensin converting enzyme inhibitors, COR, crude odds ratio, AOR, adjusted odds ratio, and CI, confidence interval.

**Table 5:** Factors associated with optimal dosing of ACEIs (n=230).

Variables	ACEIs dose		COR (95% CI)	p-value	AOR (95%CI)	p-value
	Optimal, n (%)	Suboptimal, n (%)				
Age category						

<65	69 (84.1)	97 (65.5)	1	1	1	1

≥65	13 (15.9)	51 (34.5)	2.79 (1.41-5.52)	0.003	2.61 (1.20-5.64)	0.015

Hospitalization in the last one year						

No	48 (58.5)	46 (31.1)	1	1	1	1

Yes	34 (41.5)	102 (68.9)	3.13 (1.79-5.48)	P<0.001	2.08 (1.11-3.92)	0.024

NYHA class						

I	10 (12.2)	11 (7.4)	1	1	1	1

II	48 (58.5)	77 (52)	1.51 (0.58-3.92)	0.40	1.70 (0.54-4.87)	0.395

III	24 (29.3)	60 (40.5)	2.59 (0.97-6.93)	0.057	2.27 (0.72-7.19)	0.163

Number of comorbidities						

<2	40 (48.8)	39 (26.4)	1	1	1	1

>=2	42 (51.2)	109 (73.6)	2.90 (1.64-5.11)	P<0.001	1.85 (0.96-3.60)	0.068

Valvular heart disease					.	

No	67 (81.7)	132 (89.2)	1	1	1	1

Yes	15 (18.3)	16 (10.8	0.50 (0.23-1.09)	0.082	0.54 (0.22-1.33)	0.180

Diuretic use						

No	37 (45.1)		1	1	1	1

Yes	45 (54.9)		5.94 (3.08-11.46)	P<0.001	5.60 (2.75-11.40)	P<0.001

Dose of furosemide						

≤40 mg	40 (89)	79 (60.8)				

>40 mg	5 (11)	51 (39.2)	5.17 (1.91-14.0)	0.001	9.80 (3.00-31.98)	P<0.001

NYHA, New York Heart Association, ACEIs, angiotensin converting enzyme inhibitors, COR, crude odds ratio, AOR, adjusted odds ratio, and CI, confidence interval.

## Data Availability

The dataset used to support the findings of this study is available from the corresponding author upon request.

## References

[1] Savarese G., Lund L. H. (2017). Global public health burden of heart failure. *Cardiac Failure Review*.

[2] Agbor V. N., Essouma M., Ntusi N. A. B., Nyaga U. F., Bigna J. J., Noubiap J. J. (2018). Heart failure in sub-Saharan Africa: a contemporaneous systematic review and meta-analysis. *International Journal of Cardiology*.

[3] Nyaga U. F., Bigna J. J., Agbor V. N., Essouma M., Ntusi N. A. B., Noubiap J. J. (2018). Data on the epidemiology of heart failure in Sub-Saharan Africa. *Data in Brief*.

[4] Komajda M., Böhm M., Borer J. S. (2018). Incremental benefit of drug therapies for chronic heart failure with reduced ejection fraction: a network meta-analysis. *European Journal of Heart Failure*.

[5] Chang H.-Y., Wang C.-C., Wei J. (2017). Gap between guidelines and clinical practice in heart failure with reduced ejection fraction: results from TSOC-HFrEF registry. *Journal of the Chinese Medical Association*.

[6] Ponikowski P., Voors A. A., Anker S. D., Bueno H., Cleland J. G., Coats A. J. (2016). 2016 ESC guidelines for the diagnosis and treatment of acute and chronic heart failure: the task Force for the diagnosis and treatment of acute and chronic heart failure of the European Society of Cardiology (ESC). Developed with the special contribution of the Heart Failure Association (HFA) of the ESC. *European Heart Journal*.

[7] Yancy C. W., Jessup M., Bozkurt B. (2017). 2017 ACC/AHA/HFSA focused update of the 2013 ACCF/AHA guideline for the management of heart failure: a report of the American College of Cardiology/American Heart Association Task Force on Clinical Practice Guidelines and the Heart Failure Society of America. *Journal of the American College of Cardiology*.

[8] Berthelot E., Eicher J., Salvat M., Seronde M., de Groote P. (2018). Medical inertia in the optimization of heart failure treatment after discharge and its relationship to outcome. *Health Care Current Reviews*.

[9] Sweileh W. M., Sawalha A. F., Rinno T. M., Zyoud S. H., Al-Jabi S. W. (2009). Optimal dosing of angiotensin-converting enzyme inhibitors in patients with chronic heart failure: a cross-sectional study in Palestine. *Annals of Saudi Medicine*.

[10] Tai C., Gan T., Zou L. (2017). Effect of angiotensin-converting enzyme inhibitors and angiotensin II receptor blockers on cardiovascular events in patients with heart failure: a meta-analysis of randomized controlled trials. *BMC Cardiovascular Disorders*.

[11] Swedberg K., Kjekshus J., CTS Group (1988). Effects of enalapril on mortality in severe congestive heart failure: results of the Cooperative North Scandinavian Enalapril Survival Study (CONSENSUS). *American Journal of Cardiology*.

[12] Sharpe D. N., Murphy J., Coxon R., Hannan S. F. (1984). Enalapril in patients with chronic heart failure: a placebo-controlled, randomized, double-blind study. *Circulation*.

[13] Konstam M. A., Rousseau M. F., Kronenberg M. W. (1992). Effects of the angiotensin converting enzyme inhibitor enalapril on the long-term progression of left ventricular dysfunction in patients with heart failure. SOLVD Investigators. *Circulation*.

[14] Cowie M. R., Komajda M. (2017). Quality of physician adherence to guideline recommendations for life-saving treatment in heart failure: an international survey. *Cardiac Failure Review*.

[15] Gheorghiade M., Albert N. M., Curtis A. B. (2012). Medication dosing in outpatients with heart failure after implementation of a practice-based performance improvement intervention: findings from IMPROVE HF. *Congestive Heart Failure*.

[16] Packer M., Poole-Wilson P. A., Armstrong P. W. (1999). Comparative effects of low and high doses of the angiotensin-converting enzyme inhibitor, lisinopril, on morbidity and mortality in chronic heart failure. *Circulation*.

[17] Sargento L., Simões A. V., Longo S., Lousada N., dos Reis R. P. (2016). Treatment with optimal dose angiotensin-converting enzyme inhibitors/angiotensin receptor blockers has a positive effect on long-term survival in older individuals (aged >70 Years) and octogenarians with systolic heart failure. *Drugs & Aging*.

[18] Schmidt S., Hürlimann D., Starck C. T. (2014). Treatment with higher dosages of heart failure medication is associated with improved outcome following cardiac resynchronization therapy. *European Heart Journal*.

[19] Cohen Solal A., Leurs I., Assyag P. (2012). Optimization of heart failure medical treatment after hospital discharge according to left ventricular ejection fraction: the future survey. *Archives of Cardiovascular Diseases*.

[20] Yancy C. W., Januzzi J. L., Allen L. A. (2018). 2017 ACC expert consensus decision pathway for optimization of heart failure treatment: answers to 10 pivotal issues about heart failure with reduced ejection fraction: a report of the american college of cardiology task force on expert consensus decision pathways. *Journal of the American College of Cardiology*.

[21] Yancy C. W., Jessup M., Bozkurt B. (2013). 2013 ACCF/AHA guideline for the management of heart failure: a report of the American college of cardiology foundation/american heart association task force on practice guidelines. *Journal of the American College of Cardiology*.

[22] Barywani S. B., Ergatoudes C., Schaufelberger M., Petzold M., Fu M. L. X. (2015). Does the target dose of neurohormonal blockade matter for outcome in systolic heart failure in octogenarians?. *International Journal of Cardiology*.

[23] Caldeira D., David C., Sampaio C. (2012). Tolerability of angiotensin-receptor blockers in patients with intolerance to angiotensin-converting enzyme inhibitors: A systematic review and meta-analysis. *American Journal of Cardiovascular Drugs*.

[24] Maggioni A. P., Dahlström U., Filippatos G. (2010). EURO bservational Research Programme: the Heart Failure Pilot Survey (ESC‐HF Pilot). *European Journal of Heart Failure*.

[25] Davidson B. T., Allison T. L. (2017). Improving heart failure patient outcomes utilizing guideline-directed therapy. *The Nurse Practitioner*.

[26] Bhat S., Kansal M., Kondos G. T., Groo V. (2018). Outcomes of a pharmacist-managed heart failure medication titration assistance clinic. *Annals of Pharmacotherapy*.

[27] Dunlap M. E. (2007). New studies influencing treatment of heart failure: 2006 update. *Pharmacotherapy: The Journal of Human Pharmacology and Drug Therapy*.

[28] Bungard T. J., McAlister F. A., Johnson J. A., Tsuyuki R. T. (2001). Underutilisation of ACE inhibitors in patients with congestive heart failure. *Drugs*.

[29] Rosenhek R. (2005). The controversial indications for ACE-inhibitors in valvular heart disease. *E-Journal Cardiol Pract*.

[30] Borer J. S., Sharma A. (2015). Drug therapy for heart valve diseases. *Circulation*.

[31] Kojima N., Williams J. M., Slaughter T. N. (2015). Renoprotective effects of combined SGLT2 and ACE inhibitor therapy in diabetic Dahl S rats. *Physiological Reports*.

[32] Rochon P. A., Sykora K., Bronskill S. E. (2004). Use of angiotensin-converting enzyme inhibitor therapy and dose-related outcomes in older adults with new heart failure in the community. *Journal of General Internal Medicine*.

[33] Atherton J. J., Hickey A. (2017). Expert comment: is medication titration in heart failure too complex?. *Cardiac Failure Review*.

[34] Atey T. M., Teklay T., Asgedom S. W., Mezgebe H. B., Teklay G., Kahssay M. (2018). Treatment optimization of angiotensin converting enzyme inhibitors and associated factors in ayder comprehensive specialized hospital: a cross-sectional study. *BMC Research Notes*.

[35] Hirt M. N., Muttardi A., Helms T. M., van den Bussche H., Eschenhagen T. (2016). General practitioners' adherence to chronic heart failure guidelines regarding medication: the GP-HF study. *Clinical Research in Cardiology*.

[36] Rodriguez-Cillero C., Menu D., d'Athis P. (2017). Potentially inappropriate use of furosemide in a very elderly population: an observational study. *International Journal of Clinical Practice*.

[37] Hunt S. A., Abraham W. T., Chin M. H. (2009). 2009 Focused update incorporated into the ACC/AHA 2005 guidelines for the diagnosis and management of heart failure in adults: a report of the american college of cardiology foundation/american heart association task force on practice guidelines: developed in collaboration with the international society for heart and lung transplantation. *Journal of the American College of Cardiology*.

[38] Lindenfeld J., Albert NM., Boehmer JP., Collins SP., Ezekowitz JA., Givertz MM. (2010). HFSA 2010 comprehensive heart failure practice guideline. *Journal of Cardiac Failure*.

